# Anti-Symmetric Mode Vibration of Electrostatically Actuated Clamped–Clamped Microbeams for Mass Sensing

**DOI:** 10.3390/mi11010012

**Published:** 2019-12-19

**Authors:** Lei Li, Yin-ping Zhang, Chi-cheng Ma, Can-chang Liu, Bo Peng

**Affiliations:** 1 School of Transportation and Vehicle Engineering, Shandong University of Technology, Zibo 255049, China; lleisnowflake@yahoo.com (L.L.); machch@sdut.edu.cn (C.-c.M.); liucanchang@sdut.edu.cn (C.-c.L.);; 2 State Key Laboratory of Mechanical System and Vibration, School of Mechanical Engineering, Shanghai Jiao Tong University, Shanghai 200240, China; 3 School of agricultural engineering and food science, Shandong University of Technology, Zibo 255049, China

**Keywords:** MEMS, anti-symmetric mode, bifurcation jump, parameter identification, nonlinear dynamic

## Abstract

This paper details study of the anti-symmetric response to the symmetrical electrostatic excitation of a Micro-electro-mechanical-systems (MEMS) resonant mass sensor. Under higher order mode excitation, two nonlinear coupled flexural modes to describe MEMS mass sensors are obtained by using Hamilton’s principle and Galerkin method. Static analysis is introduced to investigate the effect of added mass on the natural frequency of the resonant sensor. Then, the perturbation method is applied to determine the response and stability of the system for small amplitude vibration. Through bifurcation analysis, the physical conditions of the anti-symmetric mode vibration are obtained. The corresponding stability analysis is carried out. Results show that the added mass can change the bifurcation behaviors of the anti-symmetric mode and affect the voltage and frequency of the bifurcation jump point. Typically, we propose a mass parameter identification method based on the dynamic jump motion of the anti-symmetric mode. Numerical studies are introduced to verify the validity of mass detection method. Finally, the influence of physical parameters on the sensitivity of mass sensor is analyzed. It is found that the DC voltage and mass adsorption position are critical to the sensitivity of the sensor. The results of this paper can be potentially useful in nonlinear mass sensors.

## 1. Introduction

Doubly clamped microbeam is a common resonant element in Micro-electro-mechanical-systems (MEMS) sensors [1,2,3]. Due to their great potential and unique characteristics, microbeam resonant sensors have the advantages of small, fast, high sensitivity [4,5]. Besides, only a small amount of power is required to operate [6,7]. However, the structure nonlinearity and nonlinear electrostatic force seriously affect the performance of conventional micro-mass sensor [8,9,10]. For example, the nonlinear electrostatic force can cause shifts in their resonant frequency and lead to error of the measured mass [11]. Recently, nonlinear MEMS mass sensors have attracted attention due to their unique advantages. Firstly, the nonlinear parameter identification method of mass sensors can solve the error caused by nonlinear stiffness [12]. Besides, the sensitivity and accuracy of the sensor can be improved by using frequency stability and amplitude jump in nonlinear vibration [13]. In this paper, we study the effect of added mass on anti-symmetric mode vibration and utilize the dynamic jump motion of anti-symmetric mode to propose a new mass detection method.

The mode coupled vibration can introduce rich nonlinear phenomena into the MEMS research and reveal the mechanism of the complex dynamic behaviors [14,15,16,17,18]. Anti-symmetric response can be induced by the mode coupled vibration. Li et al. [17] presented coupled vibration behavior between second order and third order modes caused by the axial stress, which can be used to suppress large amplitude vibration and to reduce the possibility of large deflection. Kirkendall et al. [19] reported multi-stable coupled vibration between resonant modes of an electroelastic crystal plate and introduced a mixed analytical-numerical approach to provide new insight into these complex interactions. Wang et al. [20] proposed a simplified oscillation system, which consists of two beam-shaped cantilevers. The results showed the possibility of doubling the frequency response signal from the low frequency cantilever to the high-frequency cantilever based on this super harmonic synchronization. Okamoto et al. [21] used anti-symmetric vibration in two coupled GaAs oscillators to realize the high-sensitivity charge detection. In contrast to the frequency-shift detection using a single oscillator, coupled vibration allowed a large readout up to the strongly driven nonlinear response regime. Hammad et al. [22,23] presented an analytical model and closed form expressions describing the response of mechanically coupled resonant structures. Mode coupled vibration was utilized to implement an adjustable filter. Younis et al. [24] studied possibility of activating a three-to-one internal resonance between the first and second modes. Besides, Mode coupled vibration can be used to improve the frequency stability of nonlinear systems [13]. Du et al. [25] reported the experimental observations of the internal resonance in a coupled ductile cantilever system from the viewpoint of mass sensing and disclosed a frequency enhancement mechanism. Hajjaj et al. [26] used the nonlinear hardening, softening and veering phenomena to realize a bandpass filter of sharp roll off from the passband to the stopband. In general, mode coupled vibration behavior was gradually applied to improve the performance of resonators and expand the scope of MEMS applications [27]. 

A MEMS resonant mass sensor mainly realizes the detection by changing the resonant frequency and vibration amplitude of the structure caused by the adsorption of the elastic element of the sensor to the target analyzers [28]. Frequency shift tracking is the most common method [29]. Bouchaala et al. [30] obtained analytical formulations to calculate the induced resonance frequency shifts caused by the added mass. The results indicated that the detection sensitivity increases with the decrease of size. However, with the reduction of size, there are obvious nonlinear effects and complex bifurcation behaviors [31], which seriously affect the dynamic mechanical characteristics and mass detection performance of the sensor. Therefore, nonlinear mass sensors were proposed. Younis et al. [32] utilized the dynamic bifurcations to realize novel methods and functionalities for mass detection. It was noted that bifurcation-based mass detection methods provided for dramatically enhanced sensitivity and less performance deterioration due to measurement noise as compared to frequency shift-based methods [33]. Similarly, Kumar et al. [34] also detailed proof-of-concept experiments on bifurcation-based sensing. Preliminary results revealed the bifurcation-based sensing technique to be a viable alternative to existing resonant sensing methods. Hasan et al. [35] studied the intelligent adjustable threshold pressure switch. When the pressure exceeds the threshold, the system can be induced to produce amplitude jump, realizing the rapid sensing of pressure. However, the dynamic behavior near the bifurcation point is easily disturbed by the ambient noise, which affects the stability of the sensor. 

It can be concluded from the above analysis that mode coupled vibration can induce anti-symmetric modes and improve the performance of resonators, which may be beneficial to improve the accuracy and sensitivity of mass sensors [36,37,38]. Recently, most nonlinear mass sensors were realized by using the periodic saddle bifurcation of the resonant system, which greatly increases the sensitivity of the sensor. To the best of our knowledge, there are fewer quantitative results about a general analysis of nonlinear mass sensors by using anti-symmetric mode vibration. Besides, the mechanism of the effect of added mass on anti-symmetric mode vibration is not well understood. Through bifurcation analysis, the influence of added mass on transition mechanism of nonlinear jumping phenomena and complex nonlinear dynamic behaviors can be predicted, which motivates our present work. In this study we exploit the nonlinear jump phenomenon of the anti-symmetric mode to realize mass detection.

The structure of this paper is as follows. In Section 2, the Hamilton’s principle and Galerkin discretization is applied to obtain the equation of motion of a mass sensor. Then static analysis is carried out under different direct current (DC) voltage and added mass. In Section 3, the method of multiple scales is applied to produce an approximate solution. In Section 4, we analyze the physical conditions of the anti-symmetric mode vibration. Meanwhile, the effect of added mass on nonlinear dynamic behavior is considered. In Section 5, we propose the mass detection method by exploring the exploitation of amplitude jump behavior. In Section 6, the influence of DC voltage and mass adsorption position on the sensitivity of the sensor is considered. Finally, summary and conclusions are presented in the last section.

## 2. Problem Formulation 

**Figure 1:**
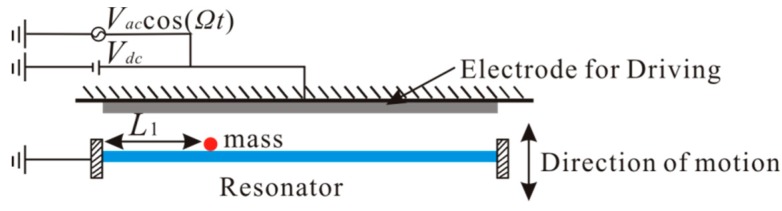
Schematic of a resonant mass sensor.

The bifurcation-based mass detection methods provide for dramatically enhanced sensitivity and less performance deterioration [32]. In our previous work, it was found that the anti-symmetric mode vibration of microbeams can be realized under the symmetrical electrostatic excitation [17]. Here, the dynamic jump behavior of anti-symmetric mode is utilized to realize mass detection. Figure 1 shows the schematic of a resonant mass sensor. The adsorption material is added to the microbeam. Then, a lumped mass *m* is added at $x=L_{1}$. The added mass can affect the equivalent mass of the system and lead to a shift in the natural frequency of the resonator. The actuation of the microbeam is realized by means of a bias voltage and an alternating current (AC) voltage component. 

By using Hamilton’s principle, the equation of motion that governs the transverse deflection $\hat{w}(\hat{x},\hat{t})$ is written as [17]

(1) $$[\rho A+\delta (x-L_{1})m]\ddot{\hat{w}}+EI{\hat{w}}^{\mathrm{iv}}+c\dot{\hat{w}}=(\frac{EA}{2L}\int_{0}^{L}{\hat{w}}^{\prime }{}^{2}dx){\hat{w}}^{\prime \prime }+\frac{\varepsilon_{0}b{\left[V_{dc}+V_{ac}\cos(\hat{\Omega }\hat{t})\right]}^{2}}{2{\left(d-\hat{w}\right)}^{2}}$$

with the boundary conditions

(2) $$\hat{w}(0,\hat{t})={\hat{w}}^{\prime }(0,\hat{t})=\hat{w}(L,\hat{t})={\hat{w}}^{\prime }(L,\hat{t})=0$$

where $\dot{\hat{w}}=\frac{\partial \hat{w}}{\partial \hat{t}}$ and ${\hat{w}}^{\prime }=\frac{\partial \hat{w}}{\partial \hat{x}}$. 

Size parameters and physical properties of resonators are listed in Table 1. The last two terms of Equation (1) represent mid-plane stretching effect and the parallel-plate electric actuation which is composed of DC and AC components. *A* and *I* represent the area and moment of inertia of the cross section. 

Then, the non-dimensional variables are introduced

(3) $$w=\frac{\hat{w}}{d},\;x=\frac{\hat{x}}{L},\;t=\hat{t}\sqrt{\frac{EI}{\rho AL^{4}}}$$

Substituting Equation (3) into Equation (1,2), yields the non-dimensional equation of motion of the mass sensor

(4) $$\ddot{w}+\eta \ddot{w}+w^{\mathrm{iv}}+c_{n}\dot{w}-(\alpha_{1}\int_{0}^{1}{w^{\prime }}^{2}dx)w^{\prime \prime }=\alpha_{2}\frac{V_{dc}^{2}}{{\left(1-w\right)}^{2}}+\alpha_{2}\frac{2V_{dc}V_{ac}\cos\Omega t+{\left(V_{ac}\cos\Omega t\right)}^{2}}{{\left(1-w\right)}^{2}}$$

with the boundary conditions

(5) $$w(0,t)=w^{\prime }(0,t)=w(1,t)=w^{\prime }(1,t)=0$$

The parameters in Equation (4) are

(6) $$\eta =\frac{\delta (x-L_{1}/L)m}{\rho AL},\;\alpha_{1}=6\times {\left(\frac{d}{h}\right)}^{2},\;\alpha_{2}=\frac{6\varepsilon_{0}L^{4}}{Ed^{3}h^{3}}$$

where η represents added equivalent mass.

The microbeam deflection under an electric force is composed of a static component due to the DC voltage, denoted by $w_{dc}(x)$, and a dynamic component due to the AC voltage, denoted by $w_{ac}(x)$; that is

(7) $$w=w_{dc}+w_{ac}$$

Ignoring the time derivatives and the AC forcing term in Equation (4), we can obtain the static deflection of the microbeam

(8) $$w_{dc}^{\mathrm{iv}}-(\alpha_{1}\int_{0}^{1}{w_{dc}^{\prime }}^{2}dx)w_{dc}^{\prime \prime }=\alpha_{2}\frac{V_{dc}^{2}}{{\left(1-w_{dc}\right)}^{2}}$$

**Figure 2:**
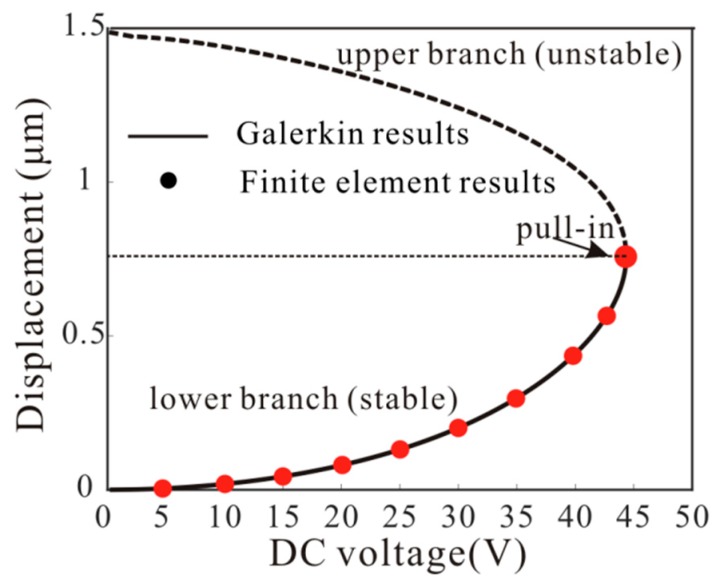
The maximum displacement of the microbeam under different voltages obtained by using the Galerkin method and COMSOL.

The static displacement of the resonator is very important to the natural frequency of the system. It was found that the nine-order mode discretization can accurately predict the static behavior of the resonator [9]. Here, the Galerkin method is introduced to calculate Equation (8). Meanwhile, the finite element method results are obtained from the software COMSOL (COMSOL Inc., Stockholm, Sweden) by using the Multi-field solver [39]. Figure 2 shows the relationship between midpoint deflections and the DC voltages obtained with the Galerkin method and the finite element method. Results are presented for values of $V_{dc}$ ranging from 0 V to pull-in voltage, where the solid line represents the position of the potential well and the dotted line represents the position of the barrier. Finite element results verify the validity of the theoretical model. It should be noted that the added mass has no effect on the static displacement.

Substituting Equation (7) into Equation (4) and using Equation (8) to eliminate the terms representing the equilibrium position, the problem governing the dynamic behavior of the microbeam around the deflected shape is generated. To third-order in $w_{ac}$, the result is

(9) $$\begin{array}{l} {\ddot{w}}_{ac}+\eta {\ddot{w}}_{ac}+c_{n}{\dot{w}}_{ac}+[w_{ac}^{\mathrm{iv}}-\alpha_{1}w_{ac}^{\prime \prime }\int_{0}^{1}{w^{\prime }}_{dc}^{2}dx-2\alpha_{1}w_{dc}^{\prime \prime }\int_{0}^{1}w_{ac}^{\prime }w_{dc}^{\prime }dx-2\alpha_{2}\frac{V_{dc}^{2}w_{ac}}{{\left(1-w_{dc}\right)}^{3}}]\\ -\alpha_{1}w_{dc}^{\prime \prime }\int_{0}^{1}{w^{\prime }}_{ac}^{2}dx-\alpha_{1}w_{ac}^{\prime \prime }\int_{0}^{1}2w_{ac}^{\prime }w_{dc}^{\prime }dx-3\alpha_{2}\frac{V_{dc}^{2}w_{ac}^{2}}{{\left(1-w_{dc}\right)}^{4}}\\ -\alpha_{1}w_{ac}^{\prime \prime }\int_{0}^{1}{w^{\prime }}_{ac}^{2}dx-4\alpha_{2}\frac{V_{dc}^{2}w_{ac}^{3}}{{\left(1-w_{dc}\right)}^{5}}\\ =2\alpha_{2}\frac{V_{dc}V_{ac}\cos\Omega t}{{\left(1-w_{dc}\right)}^{2}} \end{array}$$

Due to $V_{dc}>>V_{ac}$ [17], ${\left(V_{dc}+V_{ac}\cos\Omega t\right)}^{2}\approx V_{dc}^{2}+2V_{dc}V_{ac}\cos\Omega t$ is obtained.

The solution of Equation (9) can be expressed as $w_{ac}(x,t)=\sum_{i=1}^{\infty }u_{i}(t)\phi_{i}(x)$, where $\phi_{i}$ is the *i-th* linear undamped mode shape of the straight microbeam. Then, the linear undamped eigenvalue problem is obtained

(10) $$\phi_{i}^{iv}=(\alpha_{1}\int_{0}^{1}{w^{\prime }}_{dc}^{2}dx)\phi_{i}^{\prime \prime \prime }+\beta_{i}^{2}\phi_{i}$$

Substituting Equation (10) into the resulting Equation (9), multiplying by $\phi_{i}$, and integrating the outcome from *x* = 0 to 1, yield

(11) $$\begin{array}{l} {\ddot{u}}_{n}+\eta_{n}{\ddot{u}}_{n}+c_{n}{\dot{u}}_{n}+\beta_{n}^{2}u_{n}-\sum_{i=1}^{M}[2\alpha_{1}\int_{0}^{1}w_{dc}^{\prime \prime }\phi_{n}dx\int_{0}^{1}\phi_{i}^{\prime }w_{dc}^{\prime }dx+2\alpha_{2}V_{dc}^{2}\int_{0}^{1}\frac{\phi_{i}\phi_{n}}{{\left(1-w_{dc}\right)}^{3}}dx]u_{i}\\ -\sum_{i,j=1}^{M}\left[\alpha_{1}\int_{0}^{1}w_{dc}^{\prime \prime }\phi_{n}dx\int_{0}^{1}\phi_{i}^{\prime }\phi_{j}^{\prime }dx+\alpha_{1}\int_{0}^{1}\phi_{i}^{\prime \prime }\phi_{n}dx\int_{0}^{1}2\phi_{j}^{\prime }w_{dc}^{\prime }dx+3\alpha_{2}V_{dc}^{2}\int_{0}^{1}\frac{\phi_{i}\phi_{j}\phi_{n}dx}{{\left(1-w_{dc}\right)}^{4}}\right]u_{i}u_{j}\\ -\sum_{i,j,k=1}^{M}[\alpha_{1}\int_{0}^{1}\phi_{i}^{\prime }\phi_{j}^{\prime }dx\int_{0}^{1}\phi_{k}^{\prime \prime }\phi_{n}dx+4\alpha_{2}V_{dc}^{2}\int_{0}^{1}\frac{\phi_{i}\phi_{j}\phi_{k}\phi_{n}dx}{{\left(1-w_{dc}\right)}^{5}}]u_{i}u_{j}u_{k}\\ =f_{n}\cos\Omega t \end{array}$$

where $f_{n}=2\alpha_{2}V_{dc}V_{ac}\int_{0}^{1}\frac{\phi_{n}dx}{{\left(1-w_{dc}\right)}^{2}}$, $\eta_{n}=\phi_{n}^{2}(L_{1}/L)m/\rho AL$.

Through Equation (11), we can obtain the resonant frequency

(12) $$\omega_{n}=\sqrt{\beta_{n}^{2}-2\alpha_{1}\int_{0}^{1}w_{dc}^{\prime \prime }\phi_{n}dx\int_{0}^{1}\phi_{n}^{\prime }w_{dc}^{\prime }dx-2\alpha_{2}V_{dc}^{2}\int_{0}^{1}\frac{\phi_{n}^{2}}{{\left(1-w_{dc}\right)}^{3}}dx}$$

(13) $$\chi_{n}=\frac{1}{\sqrt{1+\eta_{n}}}\omega_{n}$$

where $\chi_{n}$ is the resonant frequency of the *n-th* order mode.

**Figure 3:**
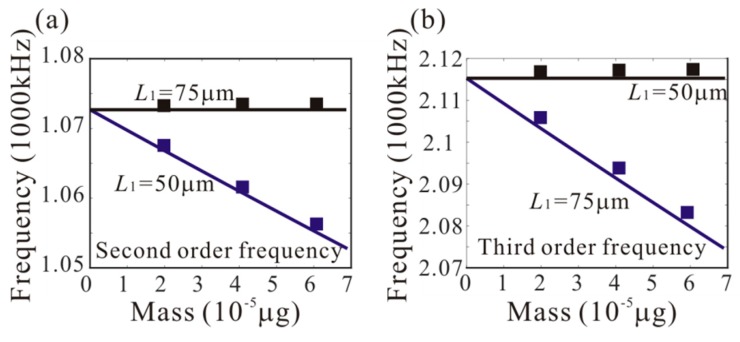
Variation of the (**a**) second and (**b**) third natural frequencies of the mass sensors with various values of adsorption position and adsorption mass (the solid lines: the theoretical results; the rectangles: COMSOL results).

**Figure 4:**
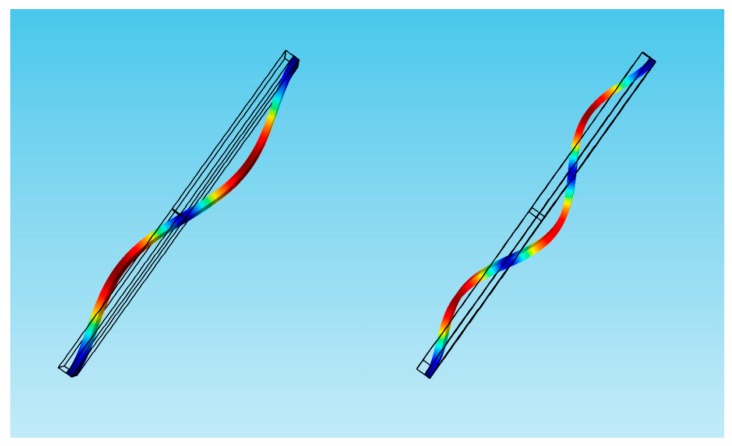
The second and third modes of the micro-mass sensor in COMSOL.

In our previous work, it was found that the third-order frequency is approximately equal to two times the second order frequency [17]. Hence we study the possibility of activating a 1:2 internal resonance between the second and third modes when the third mode is excited with a higher order excitation. Firstly, the effect of added mass on the second and third natural frequencies is considered, as shown in Figure 3. It should be noted that the effect of different adsorption positions on the natural frequency is very important. *L*_1_ = 50 µm and *L*_1_ = 75 µm are considered. When *L*_1_ = 75 µm, the added mass has a significant effect on the third modes. However, it has no effect on the second mode. Similarly, when *L*_1_ = 50 µm, the added mass has a significant effect on the second modes, and it has no effect on the third mode. To explain this phenomenon, the shape diagram of the second and third modes is obtained, as shown in Figure 4. When *L*_1_ = 75 µm, the added mass is at the node of the second-order mode. Thus, it has no effect on the second mode. Similarly, the added mass is at the node of the third-order mode under *L*_1_ = 50 µm. 

To study the dynamic behavior of the anti-symmetric mode, we take $w_{ac}(x,t)\approx \sum_{i=2}^{3}u_{i}(t)\phi_{i}(x)$ and obtain that

(14) $$\begin{array}{l} (1+\eta_{2}){\ddot{u}}_{2}+c_{n}{\dot{u}}_{2}+\omega_{2}^{2}u_{2}+a_{2r}u_{2}u_{3}+a_{2s}u_{2}^{3}+a_{2t}u_{2}u_{3}^{2}=0\\ (1+\eta_{3}){\ddot{u}}_{3}+c_{n}{\dot{u}}_{3}+\omega_{3}^{2}u_{3}+a_{3r}u_{2}^{2}+a_{3s}u_{3}^{2}+a_{3t}u_{3}^{3}+a_{3p}u_{2}^{2}u_{3}=f_{3}\cos\Omega t \end{array}$$

where the dots indicate the time derivative and the parameters are given in “Appendix A”. $\eta_{2}$ represents equivalent added mass when *L*_1_ = 50 µm; $\eta_{3}$ represents equivalent added mass when *L*_1_ = 75 µm. When the driving frequency is close to two times the natural frequency of the second order mode, the second order amplitude can produce the bifurcation jump phenomenon [17]. We take advantage of the dynamic jump behavior of the anti-symmetric mode to realize mass detection.

## 3. Perturbation Analysis 

The method of multiple scales is introduced to investigate the response of the mass sensor with small amplitude vibration. Then, ε is introduced as a small nondimensional bookkeeping parameter to indicate the significance of each term in the equation of motion. Considering the electrostatic force term $f_{3}=O(\varepsilon^{3})$, scaling the dissipative terms, we obtain

(15) $$\begin{array}{l} (1+\eta_{2}){\ddot{u}}_{2}+\varepsilon^{2}c_{n}{\dot{u}}_{2}+\omega_{2}^{2}u_{2}+a_{2r}u_{2}u_{3}+a_{2s}u_{2}^{3}+a_{2t}u_{2}u_{3}^{2}=0\\ (1+\eta_{3}){\ddot{u}}_{3}+\varepsilon^{2}c_{n}{\dot{u}}_{3}+\omega_{3}^{2}u_{3}+a_{3r}u_{2}^{2}+a_{3s}u_{3}^{2}+a_{3t}u_{3}^{3}+a_{3p}u_{2}^{2}u_{3}=\varepsilon^{3}f_{3}\cos\Omega t \end{array}$$

To describe the 1:2 internal resonance, detuning parameters δ and Δ are defined by

(16) $$\omega_{3}=2\omega_{2}-\varepsilon^{2}\Delta ,\;\Omega =\omega_{3}-\varepsilon^{2}\delta$$

Finally, the frequency response equation can be derived as

(17) $$c_{n}^{2}+{\left[(\delta +\Delta -\eta_{2}\omega_{2})+\kappa_{2}a_{3}^{2}\right]}^{2}-\frac{a_{2r}^{2}a_{3}^{2}}{4\omega_{2}^{2}}+2\kappa_{1}[(\delta +\Delta -\eta_{2}\omega_{2})+\kappa_{2}a_{3}^{2}]a_{2}^{2}+\kappa_{1}^{2}a_{2}^{4}=0$$

(18) $$\begin{array}{l} {\left(\delta -\frac{\eta_{3}\omega_{3}}{2}+\kappa_{3}a_{3}^{2}+\kappa_{4}a_{2}^{2}\right)}^{2}a_{3}^{2}+\frac{c_{n}^{2}}{4}a_{3}^{2}+{\left(\frac{a_{3r}a_{2}^{2}}{4\omega_{3}}\right)}^{2}+\frac{c_{n}^{2}}{8}a_{2}^{2}\\ -\frac{1}{4}(\delta +\Delta -\eta_{2}\omega_{2}+\kappa_{1}a_{2}^{2}+\kappa_{2}a_{3}^{2})(\delta -\frac{\eta_{3}\omega_{3}}{2}+\kappa_{3}a_{3}^{2}+\kappa_{4}a_{2}^{2})a_{2}^{2}=\frac{f_{3}^{2}}{4\omega_{3}^{2}} \end{array}$$

The detailed derivation process is given in “Appendix B”. In this paper, pseudo-trajectory processing method is introduced to solve Equations (17) and (18).

## 4. The Anti-Symmetric Mode Vibration

In this section, anti-symmetric mode vibration behaviors of MEMS mass sensors are considered. Firstly, we study the physical conditions of the anti-symmetric mode vibration.

### 4.1. Physical Conditions for Mode Transition

Anti-symmetric modes cannot be directly excited by symmetric driving forces. Mode coupled vibrations can be utilized to induce anti-symmetric modes. When the driving frequency is far away from two times the second natural frequency or the electrostatic excitation is very small, there is no second order vibration [17]. The bifurcation analysis is introduced to obtain the physical conditions of the second order vibration. When the second order amplitude occurs, we can obtain the following equation by Equation (17)

(19) $$c_{n}^{2}+{\left[(\delta +\Delta -\eta_{2}\omega_{2})+\kappa_{2}a_{3}^{2}\right]}^{2}-\frac{a_{2r}^{2}a_{3}^{2}}{4\omega_{2}^{2}}=0$$

Then, the threshold of third order amplitude can be obtained by solving Equation (19)

(20) $$a_{3}^{2}=\frac{\frac{a_{2r}^{2}}{4\omega_{2}^{2}}-2\kappa_{2}(\delta +\Delta -\eta_{2}\omega_{2})-\sqrt{{\left[\frac{a_{2r}^{2}}{4\omega_{2}^{2}}-2\kappa_{2}(\delta +\Delta -\eta_{2}\omega_{2})\right]}^{2}-4\kappa_{2}^{2}[{\left(\delta +\Delta -\eta_{2}\omega_{2}\right)}^{2}+c_{n}^{2}]}}{2\kappa_{2}^{2}}$$

Because the displacement of the second order vibration mode at the midpoint of the microbeam is always 0, the added mass has no effect on the critical amplitude when *L*_1_ = 75 µm.

When third order amplitude is more than the above threshold, the mode coupled vibration can occur. From Equation (20), the precondition of anti-symmetric mode vibration is obtained.

(21) $$\delta \leq \frac{a_{2r}^{2}}{16\kappa_{2}\omega_{2}^{2}}-\frac{4\kappa_{2}\omega_{2}^{2}c_{n}^{2}}{a_{2r}^{2}}-\Delta +\eta_{2}\omega_{2}$$

**Figure 5:**
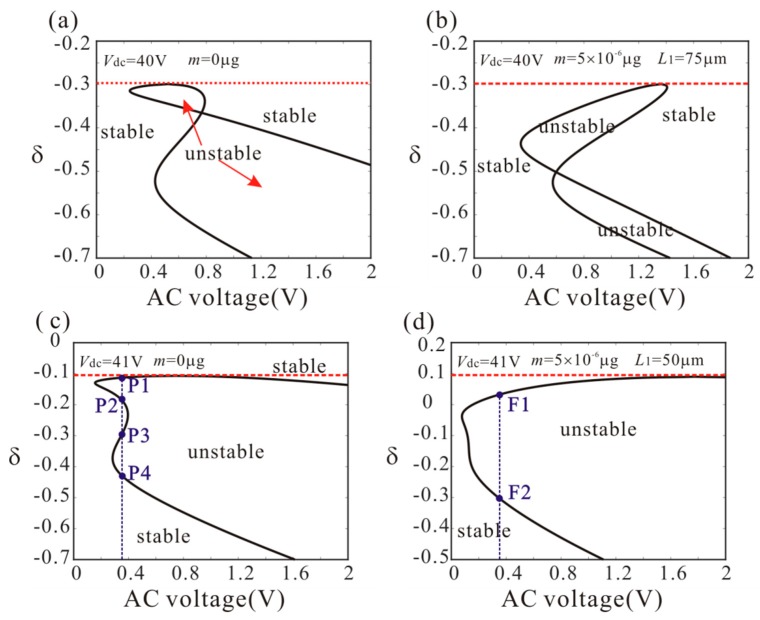
Physical conditions of anti-symmetric mode vibration under different DC voltage and added mass. (The red dotted line represents the precondition of anti-symmetric mode vibration).

When the anti-symmetric mode vibration occurs, $a_{2}=0$ becomes unstable. Then, substituting $a_{2}=0$ into Equation (17) and Equation (18), the physical conditions of anti-symmetric mode vibration under different DC voltage and added mass are obtained, as shown in Figure 5. The unstable region represents the occurrence of anti-symmetric mode vibration. It is noted that: (1) with the increase of DC voltage, the minimum critical frequency of anti-symmetric mode vibration decreases; (2) the added mass cannot affect the minimum critical frequency of anti-symmetric mode when *L*_1_ = 75 µm; (3) the added mass can decrease the minimum critical frequency of anti-symmetric mode when *L*_1_ = 50 µm; (4) the anti-symmetric mode vibration behavior depends heavily on DC voltage and added mass. Therefore, we can use the bifurcation behavior of anti-symmetric mode vibration to detect the mass.

### 4.2. Stability of the Nontrivial Solution

To further study the bifurcation behavior when the anti-symmetric mode vibration occurs, the stability analysis of the nontrivial solution is introduced. The subcritical bifurcation can lead to unstable branches near the critical points. On the contrary, the supercritical bifurcation can lead to stable branches. To determine the stability of periodic vibration, we ignore the higher-order nonlinear terms and obtain the following equation by Equation (17)

(22) $$c_{n}^{2}+{\left[(\delta +\Delta -\eta_{2}\omega_{2})+\kappa_{2}a_{3}^{2}\right]}^{2}-\frac{a_{2r}^{2}a_{3}^{2}}{4\omega_{2}^{2}}+2\kappa_{1}[(\delta +\Delta -\eta_{2}\omega_{2})+\kappa_{2}a_{3}^{2}]a_{2}^{2}=0$$

Substituting Equation (20) into Equation (22) yields the discriminant

(23) $$M=\kappa_{1}[a_{2r}^{2}(\delta +\Delta -\eta_{2}\omega_{2})+4\omega_{2}^{2}\kappa_{2}c_{n}^{2}]$$

The case M<0 results in the subcritical bifurcation. With the increase of AC voltage, the second order amplitude suddenly appeared. Likewise, the case M>0 results in the supercritical bifurcation. With the increase of AC voltage, the small vibration in the second order mode appears.

**Figure 6:**
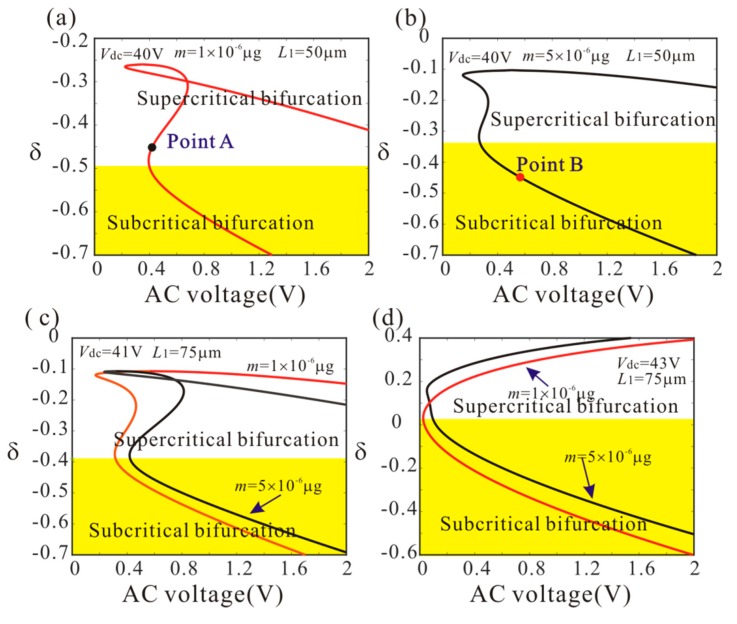
Variation of the bifurcation behavior versus δ, DC voltage and add mass. (The solid line represents the critical condition of anti-symmetric mode vibration).

Figure 6 shows variation of the bifurcation behavior versus δ, DC voltage and added mass. In the yellow area, the dynamic jump behavior of anti-symmetric mode vibration occurs with the increase of AC voltage. It is found that the increase of the DC voltage makes the dynamic jump behavior occur easily. It is interesting to note that low frequency perturbation parameter is more advantageous to realize the dynamic jump behavior than the high frequency perturbation parameter. Meanwhile, the added mass has obvious influence on the bifurcation behavior of the system when *L*_1_ = 50 µm. As the added mass increases, the critical frequency of subcritical bifurcation decreases. However, the added mass has no influence on the critical frequency of subcritical bifurcation when *L*_1_ = 75 µm.

### 4.3. Dynamic Analysis

To further study the effect of added mass on anti-symmetric mode vibration behaviors, dynamic behaviors of the anti-symmetric mode under different adsorption position and mass are introduced.

◆ *L*_1_ = 50 µm

**Figure 7:**
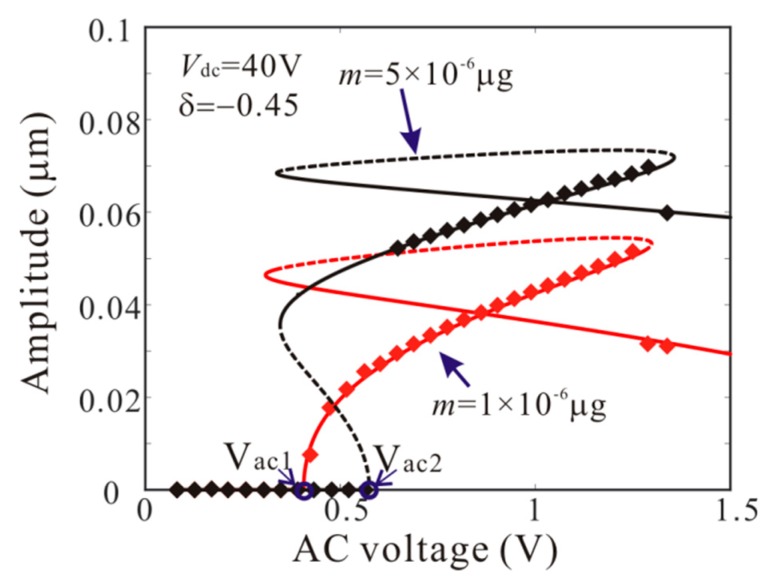
Comparison of the force-amplitude curves obtained by theoretical method (line) and numerical method (rectangle) corresponding to A-B in Figure 6 when *L*_1_ = 50 µm (solid line: stable; dashed line: unstable).

Firstly, *L*_1_ = 50 µm is considered. As shown in Figure 6, point A and point B represents supercritical bifurcation and subcritical bifurcation, respectively. Then, Figure 7 shows the force-amplitude curves corresponding to point A and point B. When $m=1\times 10^{-6}$ μg, the supercritical bifurcation voltage *V*_ac1_ is obtained. With the increase of the driving voltage, the second order amplitude appears gently. When $m=5\times 10^{-6}$ μg, the bifurcation voltage increases and the supercritical bifurcation is transformed into subcritical bifurcation. The subcritical bifurcation voltage *V*_ac2_ is obtained and the second order amplitude is suddenly generated with the increase of the driving voltage. To validate the above analysis, long-time integration (LTI) of Equation (14) is used to obtain some numerical solutions (discrete points), compared with the analytical solution derived from the method of multiple scales.

**Figure 8:**
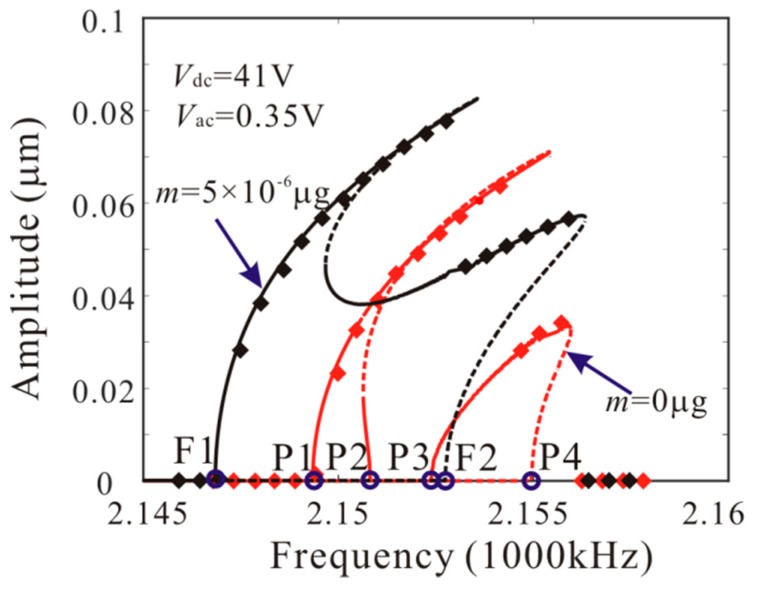
Amplitude-frequency response curves of anti-symmetric mode when *m* = 0 µg and *m* = 5 × 10^−6^ µg (line: theoretical method; rectangle: numerical method).

Then, Figure 8 shows the effect of added mass on amplitude-frequency response curve. There are four critical frequencies for anti-symmetric mode vibration behavior without considering the added mass. P1, P2, P3 and P4 indicate the bifurcation frequency of the anti-symmetric mode vibration as shown in Figure 5c. It is found that: (1) when the frequency is less than P1, no anti-symmetric mode vibration occurs; (2) when the frequency is between P1 and P2, the anti-symmetric mode vibration occurs and there is only one stable periodic solution in the system; (3) when the frequency is between P2 and P3, the trivial solution of the second order amplitude becomes stable. There are two stable and one unstable periodic solutions in the system and the second order amplitude depends on the initial conditions; (4) when the frequency is between P3 and P4, the anti-symmetric mode vibration occurs and there are two stable periodic solutions in the system; (5) when the frequency is more than P4, the anti-symmetric mode vibration behavior disappears. Then, there are two critical frequencies for anti-symmetric mode vibration behavior with considering added mass. When the frequency is between F1 and F2 (Figure 5d), the anti-symmetric mode vibration occurs and there are two stable periodic solutions in the system. F1 represents the supercritical bifurcation type and F2 represents the subcritical bifurcation type. As the drive frequency decreases, the second order amplitude can be suddenly generated when the frequency is equal to F2. From Figure 8, it is found that the added mass can change the number of critical frequencies for anti-symmetric mode vibration behavior. Meanwhile, the added mass reduces the subcritical bifurcation frequency.

◆ *L*_1_ = 75 µm

**Figure 9:**
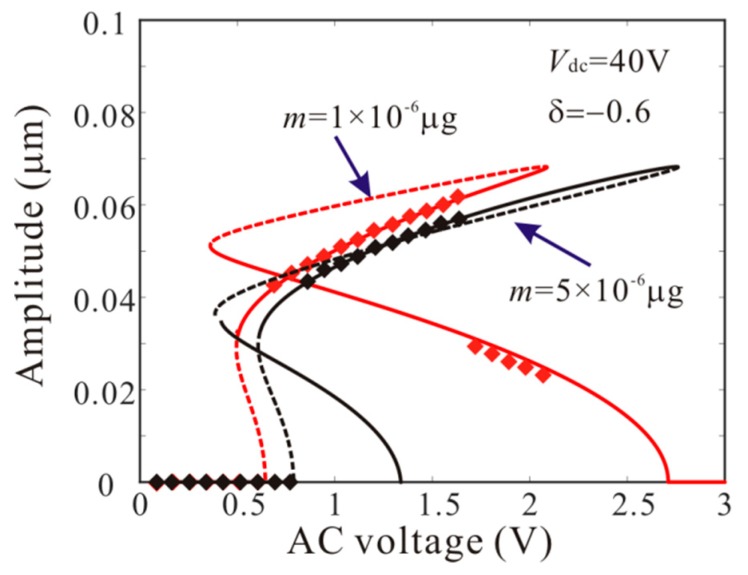
Comparison of the force-amplitude curves with considering the different added mass when *L*_1_ = 75 µm.

**Figure 10:**
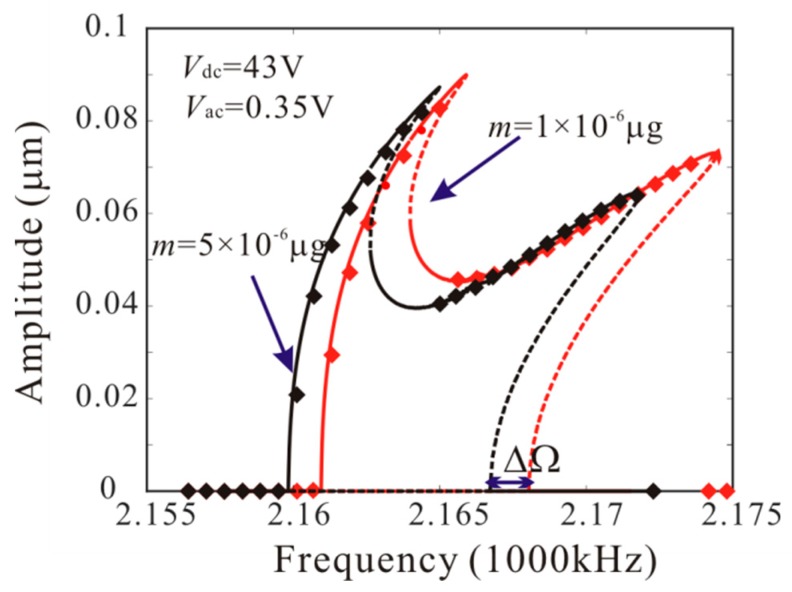
Amplitude-frequency response curve of the anti-symmetric mode considering the different added mass when *L*_1_ = 75 µm (line: theoretical method; rectangle: numerical method).

Similarly, *L*_1_ = 75 µm is considered. From Equation (23), the added mass has no effect on bifurcation type when *L*_1_ = 75 µm. However, the added mass can affect the bifurcation voltage and frequency. Figure 9 shows the force-amplitude curves obtained by pseudo-trajectory processing method (line) and long-time integration method. With the increases of added mass, the subcritical bifurcation voltage increases and the supercritical bifurcation voltage decreases. Then, amplitude-frequency response curves of the system considering the different added masses are introduced, as shown in Figure 10. There are two critical frequencies for anti-symmetric mode vibration behavior. The increase of the added mass shifts down the forced frequency response, Δ*Ω* being the subcritical bifurcation frequency shift.

When *L*_1_ = 50 µm and *L*_1_ = 75 µm, the added mass can increase the inertial forces of the second order vibration mode and the third order vibration mode, respectively. Through Figure 7, Figure 8, Figure 9 and Figure 10, the effect of added mass on anti-symmetric mode vibration behavior can be summarized as follows: (1) the added mass can adjust the number of bifurcation points near the origin; (2) the supercritical bifurcation near the origin can be transformed into subcritical bifurcation by the added mass; (3) the added mass can reduce the bifurcation frequency of the dynamic jump motion near the origin and increase the bifurcation voltage of the dynamic jump motion near the origin.

## 5. Mass Detection Method

In Section 4, we study the effect of added mass on anti-symmetric mode vibration behavior in detail. Subcritical bifurcation can lead to the dynamic jump motion, which greatly improves the sensitivity of the sensor. It is found that the added mass has an important influence on the bifurcation voltage and frequency of the dynamic jump motion. Then, we utilize of amplitude jump behavior to realize mass detection in MEMS.

First of all, the principle of mass detection is given as follow:

(1) It is found that when the driving voltage increases and the driving frequency decreases, the dynamic jump motion of the anti-symmetric mode can occur. Through the force-amplitude curve and amplitude-frequency response curve, we can obtain the bifurcation frequency and voltage.

(2) Then, we consider the mass identification formula in the case of *L*_1_ = 50 µm and *L*_1_ = 75 µm, respectively.

◆ *L*_1_ = 50 µm

From Figure 7, Figure 8, Figure 9 and Figure 10, the third order amplitude is very small when the subcritical bifurcation occurs. Thus, we can ignore the nonlinearity of the third order mode and obtain by Equation (18)

(24) $$\delta^{2}a_{3}^{2}+\frac{c_{n}^{2}}{4}a_{3}^{2}=\frac{f_{3}^{2}}{4\omega_{3}^{2}}$$

Then, substituting Equation (24) into Equation (19) yields

(25) $$\eta_{2}=\frac{1}{\omega_{2}}[\delta +\Delta +f_{3}^{2}\kappa_{2}/\left(4\omega_{3}^{2}\delta^{2}+\omega_{3}^{2}c_{n}^{2}\right)+\sqrt{a_{2r}^{2}f_{3}^{2}/4\omega_{2}^{2}(4\omega_{3}^{2}\delta^{2}+\omega_{3}^{2}c_{n}^{2})-c_{n}^{2}}]$$

◆ *L*_1_ = 75 µm

Substituting Equation (20) into Equation (18) yields

(26) $$\eta_{3}=\frac{2}{\omega_{3}}(\delta +\kappa_{3}a_{3}^{2}+\sqrt{\frac{f_{3}^{2}}{4\omega_{3}^{2}a_{3}^{2}}-\frac{c_{n}^{2}}{4}})$$

where *a*_3_ can be obtained by Equation (20).

(3) Finally, we can obtain the added mass *m* with dimensional transformation.

(27) $$m=\eta_{n}\rho AL/\phi_{n}^{2}(L_{1}/L)$$

**Figure 11:**
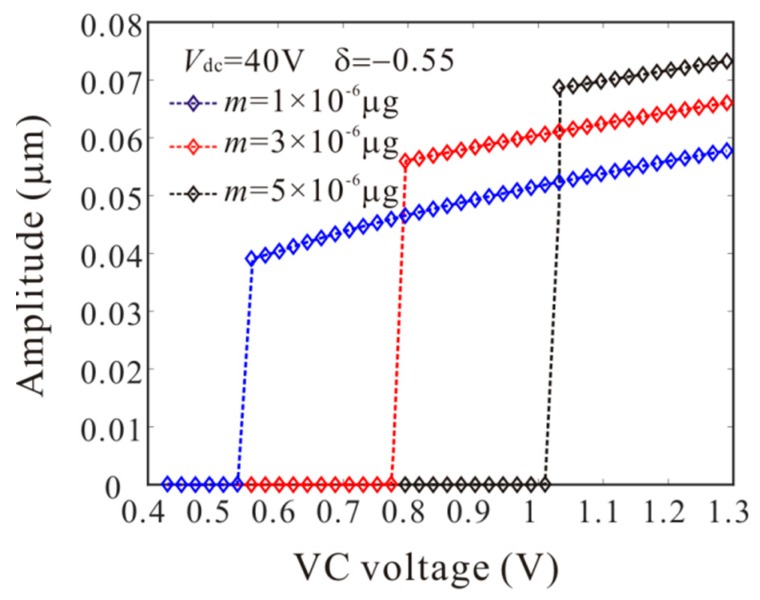
Comparison of the force-amplitude curves obtained by long-time integration method considering the different added mass when *L*_1_ = 50 µm.

**Figure 12:**
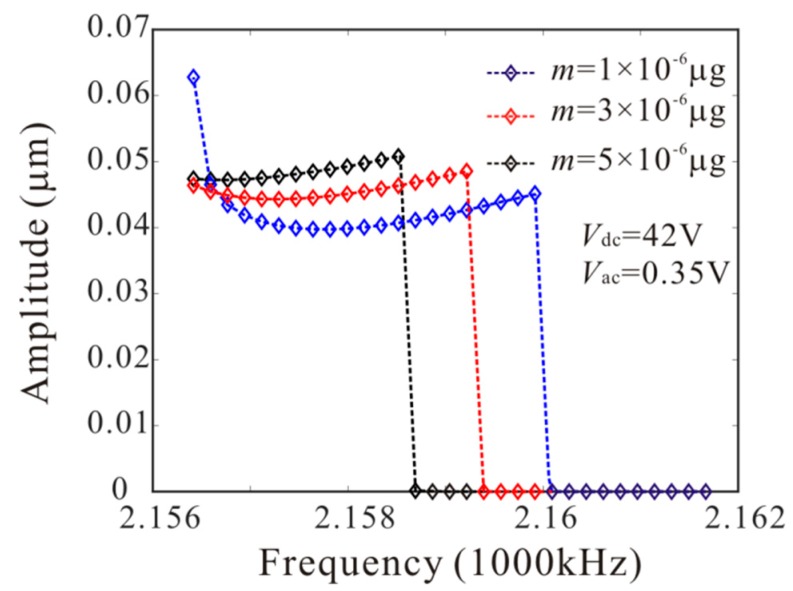
The amplitude-frequency response curve of the system considering the different added mass when *L*_1_ = 50 µm.

**Figure 13:**
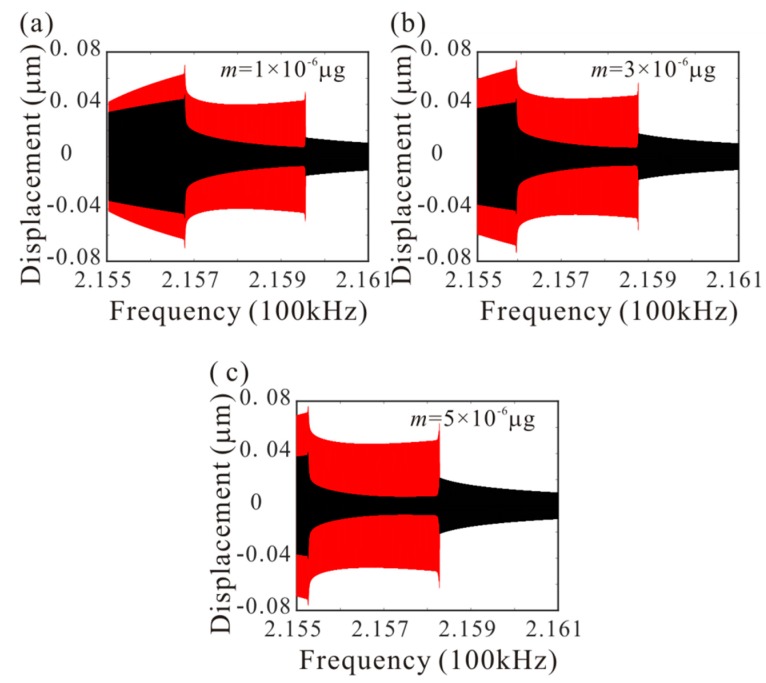
Swept harmonic responses for midpoint displacement when $V_{dc}=42$ V and $V_{ac}=0.35$ V.

**Figure 14:**
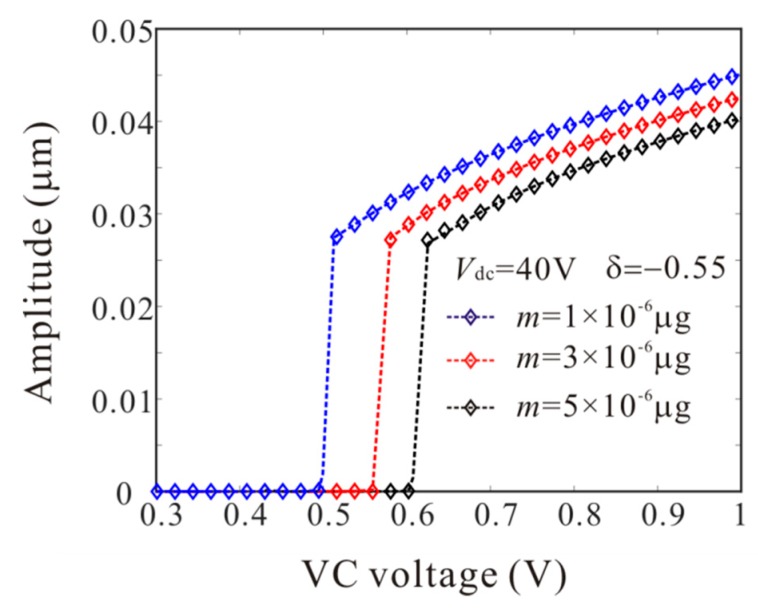
Comparison of the force-amplitude curves obtained by long-time integration method considering the different added mass when *L*_1_ = 75 µm.

**Figure 15:**
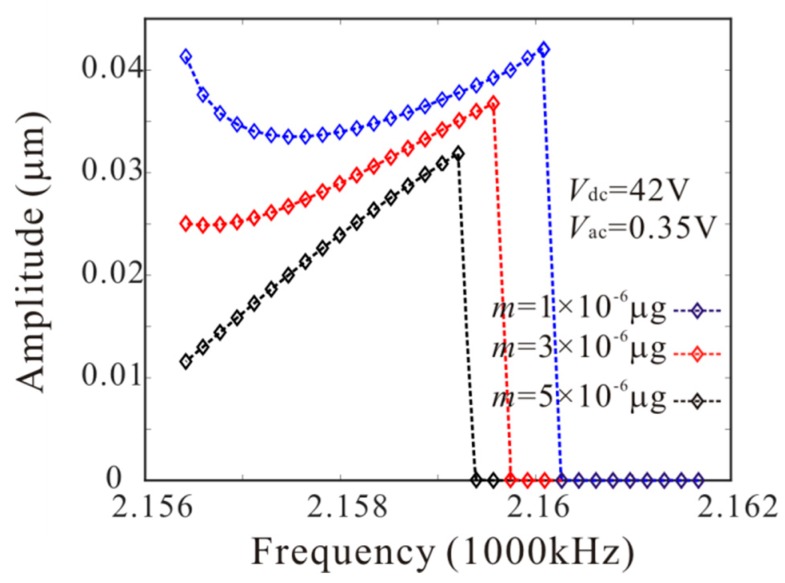
Amplitude-frequency response curve of the system considering the different added mass when *L*_1_ = 75 µm.

The numerical studies are introduced to prove the mass identification method. Firstly, *L*_1_ = 50 µm is considered. Figure 11 show force-amplitude curve obtained by sweeping up the AC voltage. As the added mass increases, the bifurcation voltage increases when δ=−0.55. With Equations (25) and (27), we obtain three kinds of parameter identification results, as shown in Table 2. The results show that the mass detection method presented in this paper can identify the added mass. Figure 12 show amplitude-frequency response curve of the system considering the different added mass obtained by sweeping down the frequency. As the drive frequency decreases, there are jump phenomena in the second order amplitude. Besides, the added mass reduces the bifurcation frequency of the jump point. Then, swept harmonic responses for midpoint displacement are introduced to further verify the bifurcation jump phenomenon, as shown in Figure 13. Here, we also obtain three kinds of parameter identification results by using Figure 12, as shown in Table 3. Similarly, Figure 14 and Figure 15 show force-amplitude curve and amplitude-frequency response curve in the case of *L*_1_ = 75 µm. With Equations (26) and (27), we obtain mass identification results, as shown in Table 4 and Table 5. There is a small error between the identification result and the real result. There are two main error sources: (1) The nonlinear stiffness terms of the third order modes are ignored when *L*_1_ = 50 µm; (2) the accuracy of numerical studies depends on the density of discrete points. An insufficient number of discrete points may lead to identification error.

## 6. Sensor Sensitivity

**Figure 16:**
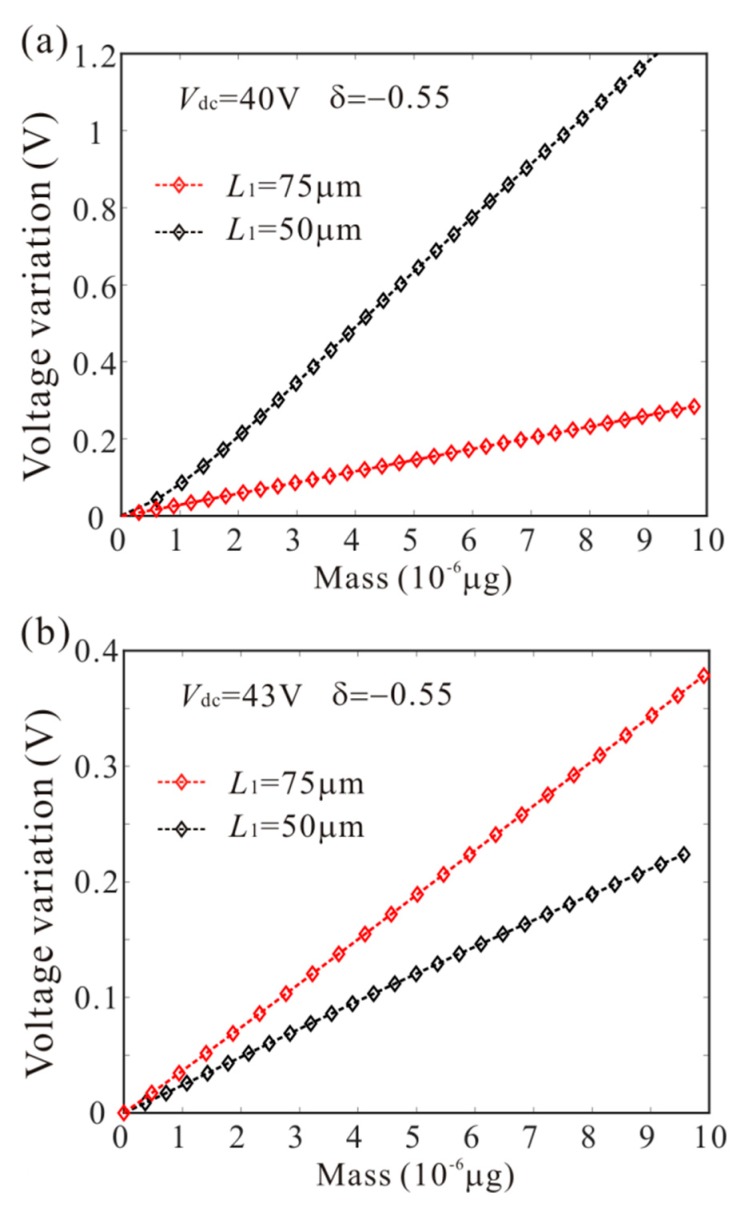
The effect of added mass at different positions on AC voltage of bifurcation jump points.

**Figure 17:**
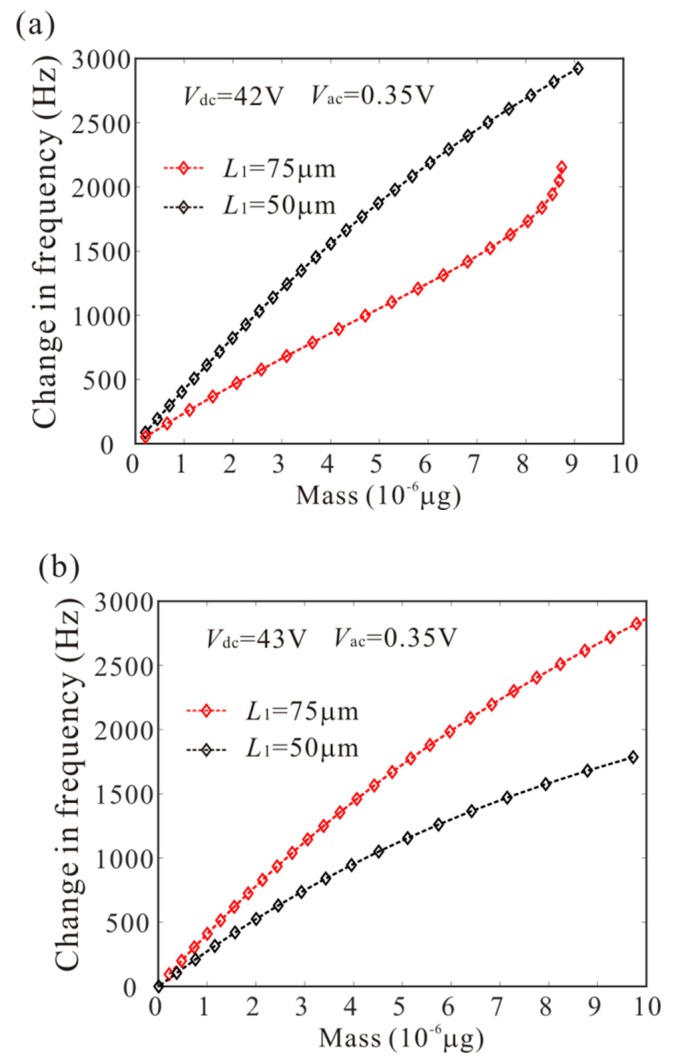
The effect of added mass at different positions on frequency of bifurcation jump points.

In this section, considering different added positions and DC voltages, we study the sensitivity of mass sensors. Variation of bifurcation voltage and bifurcation frequency caused by the added mass is introduced to characterize the sensitivity of the sensor. Figure 16 and Figure 17 show the effect of added mass at different positions on bifurcation voltage and bifurcation frequency. As the DC voltage increases, the sensitivity of the mass sensor in the case of *L*_1_ = 75 µm is improved and the sensitivity of the mass sensor in the case of *L*_1_ = 50 µm is suppressed. Thus, we need to select a reasonable DC drive voltage and adsorption position to improve the sensitivity of the mass sensor.

## 7. Conclusions

This paper studies the anti-symmetric mode vibration behavior of MEMS mass sensors, obtains the influence mechanism of added mass on anti-symmetric mode vibration behavior and utilizes the dynamic jump motion of anti-symmetric mode vibration to realize the mass detection.

Firstly, the Hamilton’s principle and Galerkin discretization are applied to obtain two degrees of freedom equations of the resonant structure. The results show that the added mass at different adsorption positions has an important effect on the natural frequency of the resonator. Through bifurcation analysis, physical conditions of anti-symmetric mode vibration behavior are obtained. The anti-symmetric mode vibration behavior depends heavily on DC voltage and added mass. Besides, it is found that the added mass can change the bifurcation type of the resonator and affect the bifurcation voltage and bifurcation frequency of the anti-symmetric mode vibration.

The dynamic jump motion is introduced to realize mass detection, which greatly improves the sensitivity of the mass sensor. The parameter identification formula of mass detection is deduced and numerical studies are used to verify mass parameter identification method. Finally, the sensitivity of the sensor is analyzed. With the increase of DC voltage, the sensitivity of the mass sensor is improved when *L*_1_ = 75 µm. On the contrary, with the increase of DC voltage, the sensitivity of the mass sensor is suppressed when *L*_1_ = 50 µm. Therefore, we need to adjust the DC drive voltage of the resonator according to different mass adsorption positions. The framework presented here provides theoretical support for nonlinear mass sensors. It should be emphasized that all the theoretical results in this paper are compared with numerical results, which guarantees the accuracy of our whole investigations.

It is worthy to mention that the proposed mass detection method discussed in this paper need to be investigated for their stability to external disturbances. Fork bifurcation occurs near the critical voltage and frequency. Hence, the stability of operation prior to mass detection must be ensured to prevent accidental bifurcation jump phenomenon due to noises or disturbances. This can be studied by conducting global dynamic analysis to track the basin of attraction of the stable solution.

## Figures and Tables

**Table 1:** Mass sensor parameters and physical properties.

Physical Parameter (units).	Value
Length of the beam electrode, *L* (µm)	150
Thickness of the beam electrode, *h* (µm)	1
Width of the beam electrode, *b* (µm)	10
Gap between the electrodes, *d* (µm)	1.5
Density of the electrode material, *ρ* (kg/m^3^)	2300
Young’s Modulus, *E* (GPa)	169
Dielectric constant of the medium, $\varepsilon_{0}$	8.85 × 10^−12^
Quality factor, *Q*	12.5

**Table 2:** Three groups of mass detection results obtained by the force-amplitude curve when $L_{1}=50\;\mu m$, δ=−0.55.

Number	The True Mass *m* (10^−6^ µg)	Bifurcation Voltage (V)	Identification Results *m* (10^−6^ µg)	Error (10^−6^ µg)
1	1	0.537	0.995	0.005
2	3	0.774	2.833	0.167
3	5	1.032	4.624	0.376

**Table 3:** Three groups of mass detection results obtained by the amplitude-frequency response curve when $L_{1}=50\;\mu m$, $V_{ac}=0.35\;V$.

Number	The True Mass *m* (10^−6^ µg)	Bifurcation Frequency (100 kHz)	Identification Results *m* (10^−6^ µg)	Error (10^−6^ µg)
1	1	21.598	0.949	0.051
2	3	21.591	2.772	0.228
3	5	21.585	4.542	0.458

**Table 4:** Three groups of mass detection results obtained by the force-amplitude curve when $L_{1}=75\;\mu m$, δ=−0.55.

Number	The True Mass *m* (10^−6^ µg)	Bifurcation Voltage (V)	Identification Results *m* (10^−6^ µg)	Error (10^−6^ µg)
1	1	0.482	1.191	0.191
2	3	0.538	3.219	0.219
3	5	0.602	5.342	0.342

**Table 5:** Three groups of mass detection results obtained by the amplitude-frequency response curve when $L_{1}=75\;\mu m$, $V_{ac}=0.35\;V$.

Number	The True Mass *m* (10^−6^ µg)	Bifurcation Frequency (100 kHz)	Identification Results *m* (10^−6^ µg)	Error (10^−6^ µg)
1	1	21.601	0.955	0.045
2	3	21.597	3.112	0.112
3	5	21.592	4.273	0.727

## Appendix A

(A1) $$a_{2r}=-[\alpha_{1}\int_{0}^{1}\phi_{2}^{\prime \prime }\phi_{2}dx\int_{0}^{1}2\phi_{3}^{\prime }w_{dc}^{\prime }dx+6\alpha_{2}V_{dc}^{2}\int_{0}^{1}\frac{\phi_{3}\phi_{2}^{2}dx}{{\left(1-w_{dc}\right)}^{4}}]$$

(A2) $$a_{2s}=-[\alpha_{1}\int_{0}^{1}{\phi_{2}^{\prime }}^{2}dx\int_{0}^{1}\phi_{2}^{\prime \prime }\phi_{2}dx+4\alpha_{2}V_{dc}^{2}\int_{0}^{1}\frac{\phi_{2}^{4}dx}{{\left(1-w_{dc}\right)}^{5}}]$$

(A3) $$a_{2t}=-[\alpha_{1}\int_{0}^{1}{\phi_{3}^{\prime }}^{2}dx\int_{0}^{1}\phi_{2}^{\prime \prime }\phi 2dx+12\alpha_{2}V_{dc}^{2}\int_{0}^{1}\frac{\phi_{2}^{2}\phi_{3}^{2}dx}{{\left(1-w_{dc}\right)}^{5}}]$$

(A4) $$a_{3r}=-[\alpha_{1}\int_{0}^{1}w_{dc}^{\prime \prime }\phi_{3}dx\int_{0}^{1}\phi_{2}^{\prime }\phi_{2}^{\prime }dx+3\alpha_{2}V_{dc}^{2}\int_{0}^{1}\frac{\phi_{2}\phi_{2}\phi_{3}dx}{{\left(1-w_{dc}\right)}^{4}}]$$

(A5) $$a_{3s}=-[\alpha_{1}\int_{0}^{1}w_{dc}^{\prime \prime }\phi_{3}dx\int_{0}^{1}\phi_{3}^{\prime }\phi_{3}^{\prime }dx+\alpha_{1}\int_{0}^{1}\phi_{3}^{\prime \prime }\phi_{3}dx\int_{0}^{1}2\phi_{3}^{\prime }w_{dc}^{\prime }dx+3\alpha_{2}V_{dc}^{2}\int_{0}^{1}\frac{\phi_{3}\phi_{3}\phi_{3}dx}{{\left(1-w_{dc}\right)}^{4}}]$$

(A6) $$a_{3t}=-[\alpha_{1}\int_{0}^{1}\phi_{3}^{\prime }\phi_{3}^{\prime }dx\int_{0}^{1}\phi_{3}^{\prime \prime }\phi_{3}dx+4\alpha_{2}V_{dc}^{2}\int_{0}^{1}\frac{\phi_{3}\phi_{3}\phi_{3}\phi_{3}dx}{{\left(1-w_{dc}\right)}^{5}}]$$

(A7) $$a_{3p}=-[\alpha_{1}\int_{0}^{1}\phi_{2}^{\prime }\phi_{2}^{\prime }dx\int_{0}^{1}\phi_{3}^{\prime \prime }\phi_{3}dx+12\alpha_{2}V_{dc}^{2}\int_{0}^{1}\frac{\phi_{2}\phi_{2}\phi_{3}\phi_{3}dx}{{\left(1-w_{dc}\right)}^{5}}]$$

## Appendix B

The general solutions of Equation (15) can be written as

(A8) $$\begin{array}{l} u_{2}=\varepsilon u_{21}(T_{0},T_{1},T_{2})+\varepsilon^{2}u_{22}(T_{0},T_{1},T_{2})+\varepsilon^{3}u_{23}(T_{0},T_{1},T_{2})\\ u_{3}=\varepsilon u_{31}(T_{0},T_{1},T_{2})+\varepsilon^{2}u_{32}(T_{0},T_{1},T_{2})+\varepsilon^{3}u_{33}(T_{0},T_{1},T_{2}) \end{array}$$

where $T_{n}=\varepsilon^{n}t$.

Substituting Equations (16) and (A8) into Equation (15) and equating coefficients of like powers of *ε* yield

(A9) $$\begin{array}{ll} O\left(\varepsilon^{1}\right): & D_{0}^{2}u_{21}+\omega_{2}^{2}u_{21}=0\\ & D_{0}^{2}u_{31}+\omega_{3}^{2}u_{31}=0 \end{array}$$

(A10) $$\begin{array}{ll} O\left(\varepsilon^{2}\right): & D_{0}^{2}u_{22}+\omega_{2}^{2}u_{22}=-2D_{0}D_{1}u_{21}-a_{2r}u_{21}u_{31}\\ & D_{0}^{2}u_{32}+\omega_{3}^{2}u_{32}=-2D_{0}D_{1}u_{31}-a_{3r}u_{21}^{2}-a_{3s}u_{31}^{2} \end{array}$$

(A11) $$\begin{array}{cc} O\left(\varepsilon^{3}\right):\;D_{0}^{2}u_{23}+\omega_{2}^{2}u_{23} & =-2D_{0}D_{2}u_{21}-D_{1}^{2}u_{21}-2D_{0}D_{1}u_{22}-c_{n}D_{0}u_{21}\\ & -a_{2r}u_{21}u_{32}-a_{2r}u_{22}u_{31}-a_{2s}u_{21}^{3}-a_{2t}u_{21}u_{31}^{2}+\eta_{2}\omega_{2}^{2}u_{21}\\ D_{0}^{2}u_{33}+\omega_{3}^{2}u_{33} & =-2D_{0}D_{2}u_{31}-D_{1}^{2}u_{31}-2D_{0}D_{1}u_{32}-c_{n}D_{0}u_{31}-2a_{3r}u_{21}u_{22}\\ & -2a_{3s}u_{32}u_{31}-a_{3t}u_{31}^{3}-a_{3p}u_{31}u_{21}^{2}+\eta_{3}\omega_{3}^{2}u_{31}\\ & +f_{3}\cos(\omega_{3}T_{0}-\delta T_{2}) \end{array}$$

The general solutions of Equation (A9) can be written as

(A12) $$\begin{array}{l} u_{21}(T_{0},T_{1},T_{2})=A_{21}(T_{1},T_{2})e^{i\omega_{2}T_{0}}+{\bar{A}}_{21}(T_{1},T_{2})e^{-i\omega_{2}T_{0}}\\ u_{31}(T_{0},T_{1},T_{2})=A_{31}(T_{1},T_{2})e^{i\omega_{3}T_{0}}+{\bar{A}}_{31}(T_{1},T_{2})e^{-i\omega_{3}T_{0}} \end{array}$$

It is convenient to express $A_{21}$ and $A_{31}$ in the polarform, $A_{21}=\frac{1}{2}a_{2}e^{i\theta_{2}}$, $A_{31}=\frac{1}{2}a_{3}e^{i\theta_{3}}$. where $a_{2}$ and $a_{3}$ indicate the second and third order amplitudes, respectively.

Substituting Equation (A12) into Equations (A10) and (A11) yields the bifurcation equations

(A13) $$\begin{array}{l} {\dot{a}}_{2}=\frac{a_{2r}a_{2}a_{3}}{4\omega_{2}}\sin\varphi -\frac{c_{n}a_{2}}{2}\\ \dot{\varphi }=\delta +\Delta -\eta_{2}\omega_{2}+\frac{a_{2r}a_{3}}{2\omega_{2}}\cos\varphi +\kappa_{1}a_{2}^{2}+\kappa_{2}a_{3}^{2}\\ {\dot{a}}_{3}=-\frac{a_{3r}a_{2}^{2}}{4\omega_{3}}\sin\varphi -\frac{c_{n}a_{3}}{2}-\frac{f_{3}}{2\omega_{3}}\sin\beta \\ \dot{\beta }=\delta -\frac{\eta_{3}\omega_{3}}{2}+\frac{a_{3r}a_{2}^{2}}{4\omega_{3}a_{3}}\cos\varphi +\kappa_{3}a_{3}^{2}+\kappa_{4}a_{2}^{2}-\frac{f_{3}}{2\omega_{3}a_{3}}\cos\beta \end{array}$$

where

$$\varphi =2\theta_{2}+\Delta t-\theta_{3},\;\beta =\delta t+\theta_{3}$$

$$\begin{array}{l} \kappa_{1}=\frac{3a_{2s}}{4\omega_{2}}-\frac{a_{2r}a_{3r}}{2\omega_{3}^{2}\omega_{2}}\\ \kappa_{2}=\frac{a_{2t}}{2\omega_{2}}-\frac{a_{2r}a_{3s}}{2\omega_{3}^{2}\omega_{2}}+\frac{a_{2r}^{2}}{32\omega_{2}^{3}-24\omega_{2}^{2}\Delta }\\ \kappa_{3}=\frac{3a_{3t}}{8\omega_{3}}-\frac{5a_{3s}^{2}}{12\omega_{3}^{3}}\\ \kappa_{4}=\frac{a_{3p}}{4\omega_{3}}-\frac{a_{3s}a_{3r}}{2\omega_{3}^{3}}+\frac{a_{2r}a_{3r}}{32\omega_{2}^{2}\omega_{3}-24\omega_{2}\omega_{3}\Delta } \end{array}$$

To determine the stability of the periodic solution, we evaluate the Jacobian matrix of Equation (A13) at $\left(a_{20},\varphi_{0},a_{30},\beta_{0}\right)$ as

$$J=\left[\begin{array}{cccc} \frac{a_{2r}a_{30}}{4\omega_{2}}\sin\varphi_{0}-\frac{c_{n}}{2} & \frac{a_{2r}a_{20}a_{30}}{4\omega_{2}}\cos\varphi_{0} & \frac{a_{2r}a_{20}}{4\omega_{2}}\sin\varphi_{0} & 0\\ 2\kappa_{1}a_{20} & -\frac{a_{2r}a_{30}}{2\omega_{2}}\sin\varphi_{0} & \frac{a_{2r}}{2\omega_{2}}\cos\varphi_{0}+2\kappa_{2}a_{30} & 0\\ -\frac{a_{3r}a_{20}}{2\omega_{3}}\sin\varphi_{0} & -\frac{a_{3r}a_{20}^{2}}{4\omega_{3}}\cos\varphi_{0} & -\frac{c_{n}}{2} & -\frac{f_{3}}{2\omega_{3}}\cos\beta_{0}\\ \frac{a_{3r}a_{20}}{2\omega_{3}a_{30}}\cos\varphi_{0}+2\kappa_{4}a_{20} & -\frac{a_{3r}a_{20}^{2}}{4\omega_{3}a_{30}}\sin\varphi_{0} & 2\kappa_{3}a_{30} & \frac{f_{3}}{2\omega_{3}a_{30}}\sin\beta_{0} \end{array}\right]$$

When all the matrix eigenvalues are negative, the system is stable; otherwise the system is unstable. Finally, the frequency response equation can be derived as

(A14) $$c_{n}^{2}+{\left[(\delta +\Delta -\eta_{2}\omega_{2})+\kappa_{2}a_{3}^{2}\right]}^{2}-\frac{a_{2r}^{2}a_{3}^{2}}{4\omega_{2}^{2}}+2\kappa_{1}[(\delta +\Delta -\eta_{2}\omega_{2})+\kappa_{2}a_{3}^{2}]a_{2}^{2}+\kappa_{1}^{2}a_{2}^{4}=0$$

(A15) $$\begin{array}{l} {\left(\delta -\frac{\eta_{3}\omega_{3}}{2}+\kappa_{3}a_{3}^{2}+\kappa_{4}a_{2}^{2}\right)}^{2}a_{3}^{2}+\frac{c_{n}^{2}}{4}a_{3}^{2}+{\left(\frac{a_{3r}a_{2}^{2}}{4\omega_{3}}\right)}^{2}+\frac{c_{n}^{2}}{8}a_{2}^{2}\\ -\frac{1}{4}(\delta +\Delta -\eta_{2}\omega_{2}+\kappa_{1}a_{2}^{2}+\kappa_{2}a_{3}^{2})(\delta -\frac{\eta_{3}\omega_{3}}{2}+\kappa_{3}a_{3}^{2}+\kappa_{4}a_{2}^{2})a_{2}^{2}=\frac{f_{3}^{2}}{4\omega_{3}^{2}} \end{array}$$
